# HRSA-Funded MCH Pipeline Training Program: Advancing the MCH Pipeline and Workforce Through Research Collaborations

**DOI:** 10.1007/s10995-022-03439-w

**Published:** 2022-07-07

**Authors:** Omonike A. Olaleye, Deepa Dongarwar, Hamisu M. Salihu, Sylvia Adu-Gyamfi, Manvir Kaur, Anuoluwapo Egbejimi, Victoria A. Moerchen, Harolyn M. E. Belcher, Faye Holmes, Alice Kuo, Nikeea Copeland-Linder, Charlotte A. Noble, Cheryl A. Vamos, Catrina R. Waters, Claudia M. Brown, Madhavi M. Reddy

**Affiliations:** 1 Texas Southern University, 3100 Cleburne St. Houston, TX USA; 2 Baylor College of Medicine, 3701 Kirby Drive, Suite 600, Houston, TX 77098 USA; 3 University of Wisconsin-Milwaukee, Milwaukee, USA; 4 Kennedy Krieger Institute, Baltimore, MD USA; 5 University of California, Los Angeles, Los Angeles, USA; 6 University of North Texas Health Science Center, Fort Worth, USA; 7 University of South Florida, Tampa, USA; 8 Alabama State University, Montgomery, USA; 9 U.S. Department of Health and Human Services, Health Resources and Services Administration, Maternal and Child Health Bureau, MD Rockville, USA

**Keywords:** Maternal and child health, MCH, Pipeline program, Research collaborations, Mentorship

## Abstract

**Purpose:**

Presently, there are six undergraduate HRSA-funded MCH pipeline training programs (MCHPTP) in the nation and they have gained significant momentum since inception by recruiting, training and mentoring undergraduate students in a comprehensive MCH-focused approach. This article describes the outcomes from the 6 training programs; and primarily Baylor College of Medicine–Texas Southern University (BCM–TSU’s) collaborative strategy focusing on the MCH research training and outcomes, which align with HRSA’s MCH bureau’s missions.

**Description:**

Each MCHPTP offers trainees interdisciplinary MCH research experiences through intra/inter-institutional collaborations and partnerships, but BCM–TSU’s MCHPTP was the only one with the primary focus to be research. As a case study, the BCM–TSU Program developed an innovative research curriculum integrated with MCH Foundations Course that comprised 2 hour weekly meetings. Students were split into collaborative research groups of 4–5 students, with multidisciplinary peer-mentors, clinical fellows and MCH research faculty from institutions at the world—renowned Texas Medical Center.

**Assessment:**

Since the inception of the MCH mentorship programs, all six MCHPTPs have enrolled up to 1890 trainees and/or interns. BCM–TSU Program trainees are defined as undergraduate students in their 1st or 2nd year of college while research interns are upper classmen in their 3rd or 4th year of college. The case study showed that BCM–TSU Program trainees demonstrated outstanding accomplishments in the area of research through primary and co-authorships of 13 peer-reviewed journal publications by 78 trainees, over a period of 3 years, in addition to dozens of presentations at local, regional and national conferences.

**Conclusions:**

The research productivity of students in the six MCHPTPs is strongly indicative of the success of integrating MCH research mentoring into MCH didactic training. The development of a diverse and robust MCH mentorship program promotes and strengthens research activities in areas of high priority such as addressing health disparities in MCH morbidity and mortality in the U.S.

## Significance Statement

*What is already known on this subject*? The diminishing pipeline of students from underrepresented groups in the Maternal and Child Health (MCH) workforce and graduate student pool calls for early engagement of these populations in undergraduate MCH training to build a more culturally representative workforce.

*What this study adds*? From the BCM–TSU MCH pipeline training program (MCHPTP) findings, the research outcomes of the students are strongly suggestive of the success of integrating MCH research mentoring into MCH didactic training.

## Purpose

One of the most prominent features of the United States’ (U.S.’) demographics is its racial and ethnic diversity. The racial and ethnic composition of the U.S. has been changing substantially for the last few decades prompting demographic experts to predict that no singular racial/ethnic group will constitute a majority by the end of the current century (Berglas & Lim, 1998). With the U.S. being tagged the nation of immigrants, it is estimated that within the next 40 years, there will be about 70 million foreign-born people living in the U.S. (Vespa et al., 2018). Most people migrating to the U.S. belong to racial/ethnic minority groups (Vespa et al., 2018). Given this background, it is important that attention is focused on improving equity in access to health care to address emerging health disparities that disproportionately and adversely affect racial/ethnic underrepresented and underserved population. Despite recent progress, underrepresented populations continue to make up a small fraction of the health care workforce: 9% of nurses, 6% of physicians, and 5% of dentists, although these groups already account for more than a quarter of the population in the United States, a figure that is expected to rise to more than half by 2050 (Kuo et al., 2015).

Despite improvement in government interventions such as increased allocation of funds and resources to maternal and child health (MCH) care as well as remarkable medical breakthroughs over the decades, huge gaps remain in terms of effective approaches to resolve health care system failures (e.g., healthcare access, delivery of quality care) as well as in enhancing services received by the nation’s minority communities (2018 National Healthcare Quality and Disparities Report | Agency for Healthcare Research and Quality, (2022); Berglas & Lim, 1998). This will require a strong, well-coordinated and integrated public health response in order to dampen or eliminate the resulting health disparities including within the MCH population. A well-established factor that contributes to the sustenance of racial/ethnic disparities in MCH health indices is the disproportionate representation of underrepresented and underserved groups in the MCH workforce (Bozlak et al., 2022; Mishkin et al., 2021; National Research Council, 2003). The current inadequate representation of underrepresented groups in the MCH workforce poses significant cultural and linguistic barriers in the course of patient-provider interactions, providers’ intentional and unintentional biases towards patients of different backgrounds, and providers’ clinical uncertainty when treating diverse populations (Bouye et al., 2016; Phillips and Malone, 2014; Oh et al., 2015). Therefore, it is key that academic institutions recruit, engage and train students belonging to racial/ethnic underrepresented populations, and those from disadvantaged backgrounds in order to have a more effective MCH workforce capable of addressing contemporary challenges of health equity and racial/ethnic health disparities (Bouye et al., 2016; Bozlak et al., 2022).

The Division of Maternal and Child Health Workforce Development (DMCHWD), which is part of the Health Resources and Services Administration’s (HRSA’s) Maternal and Child Health Bureau (MCHB), aims for all children, youth, and families to be able to live and thrive in healthy communities served by quality workforce that helps assure their health and well-being (“Diversity and Health Equity in the Maternal and Child Health Workforce”, 2016).

To address the low representation of underrepresented individuals within the MCH workforce, HRSA developed the HRSA-funded MCH mentorship training program which aims “to promote the development of a culturally diverse and representative health care workforce by recruiting undergraduate students from economically and educationally disadvantaged backgrounds (African Americans, Hispanics/Latinos, Asian, Hawaiian/Pacific Islanders and American Indian/Alaskan Natives) into MCH professions” (Brown & Flattau, 2009). The program seeks to educate, mentor, guide, and provide enriching experiences to increase students’ interests in MCH public health professions. Currently, there are six HRSA-funded MCH pipeline training programs (MCHPTP) in the nation located at Alabama State University, Baylor College of Medicine–Texas Southern University (BCM–TSU), Kennedy Krieger Institute-Johns Hopkins University (KKI–JHU), University of California at Los Angeles (UCLA), University of South Florida (USF), and University of Wisconsin at Milwaukee (UW). Scholars at each site are trained in MCH didactics, clinical practice, research, and/or community engagement and advocacy.

A significant part of the undergraduate scholars’ training involves awareness of the multitudes of health issues confronting the MCH population. To further this philosophy, students’ involvement in MCH-related research activities and the dissemination of findings is variably regarded as an important component within each program, and as a tool that enhances students’ critical thinking and communication skills. Through this process, it is anticipated that students will be well prepared for graduate studies and integration into the ever evolving MCH workforce where these skills are vital in finding solutions to simple as well as complex challenges. The aims of this paper are twofold: (1) to describe the experience and outcomes from the MCH research activities and collaborations undertaken by underrepresented and underserved undergraduate students and professors working in the MCHPTP at BCM–TSU; and (2) to describe the lessons learned from the other MCHPTP throughout the country.

## Description

The MCHPTP at BCM–TSU is called MCH Leadership, Education, and Advancement in Undergraduate Pathways Training Program (LEAP), and we will be using the two designations interchangeably. We established a framework for the MCH LEAP program with the following components—(1) academic program quality and research—which involves creating an integrated curriculum and an innovative MCH course design and development strategy; (2) collaborative culture between faculty and students—this encompasses student engagement and advising, mentoring, training and assessment of students by the faculty; (3) partnerships—this relates to inter/intra-institutional collaborations and partnerships; (4) finance, resources and infrastructure—which is being offered through the MCH award and HRSA support; and (5) student success and completion—this relates to student recruitment, enrollment, retention, progression and graduation. A source of motivation for the integration of MCH research training and mentoring in the BCM–TSU program was the overwhelming request from the MCH undergraduate trainees, who compellingly advanced the argument that such an integration coupled with an objective product (e.g., a publication) will significantly boost their competitiveness in the MCH workforce as well as for graduate admission subsequently.

We created a four-pronged approach in developing a research and mentoring strategy for the MCH undergraduate students and their faculty mentors within the BCM–TSU collaborative. The goals were—(1) to recruit and retain a cohort of undergraduate students each year over 5 consecutive years in an MCH-focused training program that incorporates research methodology into didactic courses; (2) to train the enrolled undergraduate students in MCH core competencies using a blended didactic training approach which integrates traditional methods with cutting- edge technology (MCH lab) that creates a captivating learning environment for the undergraduate students; (3) to develop, together with input from trainees, an effective and enriching mentoring program that will meet the learning and skill development needs of the students as they navigate through the mentorship program and consider future career options in MCH-related fields; (4) to offer trainees of the MCH LEAP an inter-professional experience through participation and engagement in curricular and extracurricular activities that offer opportunities for inter- professional interactions including participation in multi-disciplinary research activities as well as dissemination of research findings at various avenues. This was the novel, innovative MCH foundation which integrated scores with cutting-edge research methodologies.

The BCM–TSU Program developed an innovative research curriculum integrated with MCH Foundations Course, which is presented here as a case study. The course consisted of a year-long training program which consisted of 2 h weekly meetings—1 h for didactic sessions and another for research training. Students were split into collaborative research groups of 4–5 students, with multidisciplinary MCH research faculty, mentors and clinical fellows from institutions at the world-renowned Texas Medical Center.

Community Health Analytics Training, or CHAT, a copyrighted digital platform was utilized to disseminate didactic, experiential and research instructions as well as personalized mentoring to the BCM–TSU MCH students (“Online Training in Community Health Analytics”, 2022). This platform allowed for monitoring students’ progress and reporting. The personalized mentoring was done through the copyrighted web-based mentoring tool, the Digital Mentoring Database, or DMD, which matched mentees to their mentors based on their subjects of interest.

We also obtained information on experiential and student-focused research activities from all the six awardees using a survey questionnaire containing the following query items: (1) How many students received admission to MCH related/Public Health graduate programs? (2) How many students entered MCH internship placements and/or workforce? (3) How many students published in peer-reviewed journals? (4) How many publications does your MCHPTP have? (5) How many students submitted abstracts and presented at local/regional/national scientific conferences? (6) How many total abstracts submissions and/or presentations has your MCHPTP participated in at local/regional/national scientific conferences? (7) How many students in total have been enrolled in your MCHPTP since inception? (8) When did your MCHPTP start (i.e. what year did you receive the grant)? (9) How many undergraduate research programs/projects have your MCHPTP offered students?

## Assessment

**Fig. 1:**
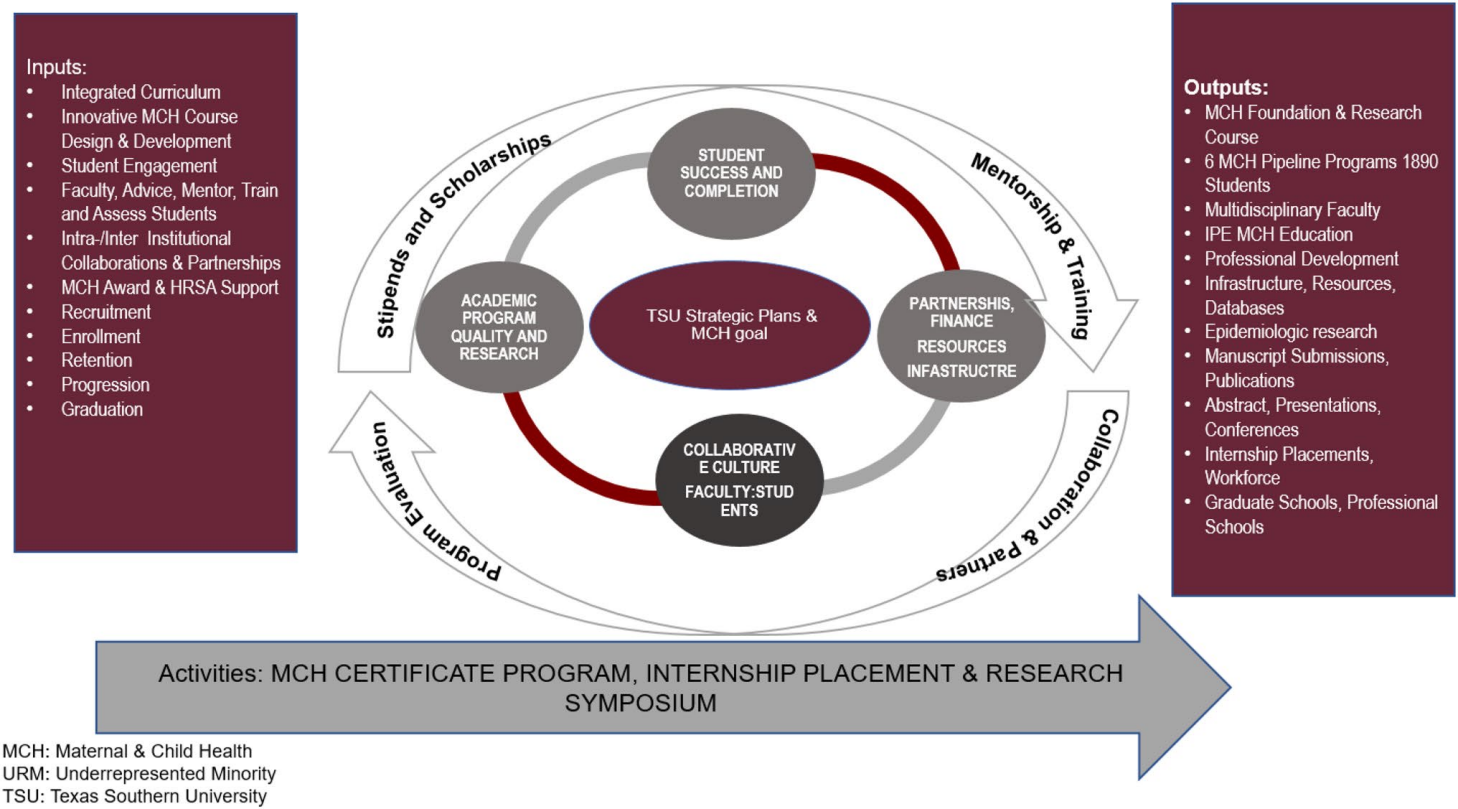
Logic model for designing an undergraduate MCH research program

A hallmark of the design of the BCM–TSU undergraduate MCH program as illustrated in Fig. 1 which shows comprehensive integration of didactic sessions with skill-building and hands-on research activities that culminated in dissemination products including manuscripts submitted and published in impactful scientific journals, oral and poster presentations at regional as well as national conferences. Over the lifetime of the grant, the BCM–TSU program developed a robust technology-based infrastructure and virtual resources that seamlessly facilitate the acquisition of MCH knowledge and research-based competencies. Examples of this infrastructure and resources include Digital Mentoring Database (DMD), BIG DATA used for the aforementioned manuscript development and publications as well as MCH LEAP ‘Research Readiness’ (R^2^), which are periodic shuttles of weekly quizzes on MCH leadership knowledge and skills. These weekly quizzes challenge the critical thinking of our MCH students and refine their MCH knowledge base as well as skill proficiency.

Another accomplishment of the MCH LEAP program is the approval by the State of Texas Higher Education Coordinating Board of our “MCH Foundation Course” which incorporates pedagogic learning and research skills acquisition, and the course is open to all students. It is.

anticipated that this course will lead eventually to the establishment of an undergraduate MCH Public Health Program in the near future. Because the MCH LEAP program at BCM–TSU had a 1-year duration, we were able to accept new students each year. About 20 students per year, mentored by 9 faculties achieved remarkable success, which was demonstrated through the 99% passing rate at the MCH LEAP assessment (1 out of 100 students did not pass) and by obtaining their MCH LEAP certification.

Additionally, the students from the BCM–TSU program created an organization called Global Alliance for Maternal and Child Health, or GLAM. All research projects and internships were domestic, however, the student leaders had the vision of expanding their organization globally, and therefore utilized the word Global in GLAM.

Table 1 shows the cumulative research outcomes among all the undergraduate MCHPTP since their respective inceptions till the end of 2019. The information was obtained using a one-time web-based survey conducted among the program directors who provided the data on all participants since the program’s inception at their respective sites. The inaugural MCHPTP grants were awarded to Alabama State University, UCLA, and UW in the year 2006. Since then the number of such programs has risen to six by 2019, an increase of 100%. The current MCHPTP at BCM–TSU, the University of South Florida and Kennedy Krieger Institute commenced in 2016. Since its inception, the BCM–TSU program has had 100 students participate in the program. The total for all grantee programs is 1907 students. Within a span of 4 years, students in the BCM–TSU program took part in 17 MCH-related projects that led to peer-reviewed journal articles (Table 2).

**Table 1:** Research outcomes among BCM–TSU and other MCHPTP

Questions	Alabama	UCLA	Kennedy Krieger-JHU N = 26	USF	UWisconsin–Milwaukee	TSU–BCM	Total
(1) How many students received admission to MCH related/Public Health graduate programs?	123	109	16	9	140	22	419
(2) How many students entered MCH internship placements and/or workforce?	76	109	21	14	130	20	370
(3) How many students published in peer-reviewed journals?	0	0	4	0	12	78	94
(4) How many publications does your MCHPTP have?	3	107	4	0	21	17	152
(5) How many students submitted abstracts and presented at local/regional/national scientific conferences	1	13	43	23	49	80	209
(6) How many total abstracts submissions and/or presentations have your MCHPTP participated in at local/regional/national scientific conferences	5	13	109	23	37	13	200
(7) How many students in total have been enrolled in your MCHPTP since inception?	216	1372	43	36	140	100	1907
(8) When did your MCHPTP start i.e. what year did you receive the grant?	2006	2006	2016	2016	2006	2016	n/a
(9) How many undergraduate research programs/projects have your MCHPTP offered students?	2	13	43	35	49	20	162

**Table 2:** List of student publications resulting from the BCM–TSU MCHPTP LEAP up till December, 2021

Author listing	Title	Journal Name and DOI	Year of publication	Outcomes
Alexander J*, Dongarwar D, Varnado L*, Adenote A*, BaileyJ*, Ezeudu C*, Nelson P*, Shavers A*, Telufusi A*, Spooner KK, Salemi JL, Salihu HM, Olaleye OA.	Temporal Trends of Gestational Malaria in the United States	Parasite Epidemiology and Control. DOI: 10.1016/j.parepi.2020.e00191	2020	Published
Kiydra Harris, Deepa Dongarwar MS, Tasha Roshan^*^, Collins Onyenaka^*^, Collins Enwerem^*^, Omonike Olaleye, Hamisu M. Salihu, MD, PhD	The Global Alliance for Maternal and Child Health (GLAM: a Pioneer Organization for MCH Students	Int J MCH AIDS DOI: 10.21106/ijma.463	2020	Published
Dongarwar D, Taylor J, Anene N*, Argueta E*, Au T*, Giger D*, Ogba C*, Oluwatoba A*, Omoyele O*, Spooner KK, Salemi JL, Olaleye OA, and Salihu HM	Trends in Appendicitis Among Pregnant Women, the Risk for Cardiac Arrest, and Maternal-Fetal Mortality	World J Sur DOI: 10.1007/s00268-020-05717-6	2020	Published
Nguyen NH*, Le EN*, Mbah VO*, Welsh EB*, Daas RO*, Spooner KK, Salemi JL, Olaleye OA, and Salihu HM.	Opioid Use Among HIV-Positive Pregnant Women and the Risk for Maternal-Fetal Complications	South Med J DOI: 10.14423/SMJ.0000000000001105	2020	Published
Salihu HM, Dongarwar D, Ikedionwu CA, Shelton A, Jenkins CM, Onyenaka C*, Charles C, Wang H, Osemene I, Harris KJ, Kaur M*, Rasmus M, Awosemo O*, Milton S, Estill S, Adebusuyi T*, Gao X*, Mbye YFN, Chen Y*, Olaleye OA.	Racial/Ethnic Disparities in the Burden of HIV/Cervical Cancer Comorbidity and Related In-hospital Mortality in the USA	J Racial Ethn Health Disparities DOI: 10.1007/s40615-020-00751-5	2020	Published
Darlington F*, Acha BM*, Roshan T*, Ikeanyionwu C*, Kutse S*, Abajue U*, Osazuwa B*, Gomez I*, Spooner KK, Salemi JL, Dongarwar D, Olaleye OA, Salihu HM, Ndefo UA.	Opioid-Related Disorders Among Pregnant Women with Sickle Cell Disease and Adverse Pregnancy Outcomes	Pain Med DOI: 10.1093/pm/pnaa188	2020	Published
Ikedionwu C, Dongarwar D, Kaur M*, Nunez L*, Awazi A*, Mallet J*, Kennedy K*, Cano M*, Dike C*, Okwudi J*, Stewart J*, Igwegbe *, Estes F, Spooner K, Salemi J, Salihu H, Olaleye O.	Trends and Associated Characteristics for Chagas Disease Among Women of Reproductive Age in the United States, 2002 to 2017	Parasite Epidemiology and Control. DOI: 10.1016/j.parepi.2020.e00167	2020	Published
Dongarwar D, Ajewole VB, Oduguwa E, Ngujede A, Harris K, Ofili, TU, Olaleye OA, Salihu HM	Role of Social Determinants of Health in Widening Maternal and Child Health Disparities in the Era of Covid-19 Pandemic	Int J MCH AIDS DOI: 10.21106/ijma.398	2020	Published
Falana A*, Akpojiyovwi V*, Sey E*, Akpaffiong A*, Agumbah O*, Chienye S*, Banks J*, Jones E*, Spooner KK, Salemi JL, Olaleye OA, and Salihu HM.	Hospital Length of Stay and Cost Burden of HIV, Tuberculosis, and HIV-Tuberculosis Coinfection Among Pregnant Women in the United States	Am J Infect Control DOI: 10.1016/j.ajic.2017.09.016	2018	Published
Dennis E*, Hao Y*, Tamambang M*, Roshan T*, Gatlin K*, Diallo F*, Ogunyemi O*, Bghigh H*, Spooner KK, Salemi JL, Olaleye OA, Salihu HM	Tuberculosis During Pregnancy in the United States: Racial/Ethnic Disparities in Pregnancy Complications and in-Hospital Death	PLoS One DOI: 10.1371/journal.pone.0194836	2018	Published
Fernandez D*, Salami I*, Davis J*, Mbah F*, Kazeem A*, Ash A*, Babino J*, Carter L*, Salemi JL, Spooner KK, Olaleye OA, and Salihu HM.	HIV-TB Coinfection among 57 Million Pregnant Women, Obstetric Complications, Alcohol Use, Drug Abuse, and Depression	J Pregnancy DOI: 10.1155/2018/5896901	2018	Published
Prophet J*, Kelly K*, Domingo J*, Ayeni H*, Djoko Mekouguem XP*, Dockery *B, Allam F*, Kaur M*, Artis J*, Spooner KK, Salemi JL, Olaleye OA, Salihu HM.	Severe Pre-Eclampsia Among Pregnant Women with Sickle Cell Disease and HIV	Pregnancy Hypertens. DOI: 10.1016/j.preghy.2018.01.006	2018	Published
Deepa Dongarwar, Veronica B Ajewole, Kiydra Harris, Emmanuella Oduguwa, Theresa U. Ofili, Collins Onyenaka^*^, Sade Arnold*, Jorhn Broussard^*^, Joan Ishioye^*^, Jasmine Marshall*, Jamila Mayoya^*^, Danchau Le^*^, Mouch Fadel^*^, Omonike A. Olaleye, Hamisu M. Salihu	A Framework for Protecting Pregnant Women in the Era of COVID-19 Pandemic	Int J MCH AIDS DOI: 10.21106/ijma.419	2021	Published
Chioma A Ikedionwu, Deepa Dongarwar, Courtney Williams*, Evelyn Odeh*, Maylis Peguy Nkeng Peh*, Hilliary Hooker*, Stacey Wiseman*, Tramauni Brock*, Erinn Payne-Green*, Chidinma Chukwudum*, Grace Loudd*, Andrea Shelton, Jonnae O Atkinson, Kiara K Spooner, Jason L Salemi, Hamisu M Salihu, Omonike A Olaleye	Trends and Risk Factors for Leishmaniasis among Reproductive Aged Women in the United States	Int J MCH AIDS DOI: 10.21106/ijma.478	2021	Published
Kiydra Harris, Deepa Dongarwar, Tasha Roshan*, Collins Onyenaka*, Collins Enwerem*, Omonike Olaleye, Hamisu M Salihu	The Global Alliance for Maternal and Child Health (GLAM): A Pioneer Organization for MCH Students	Int J MCH AIDS DOI: 10.21106/ijma.463	2021	Published
Veronica B Ajewole, Ahone E Ngujede, Emmanuella Oduguwa, Deepa Dongarwar, Manvir Kaur*, Cecelia Knight*, Maresha Jackson*, Uyen Nguyen*, Tasha Roshan*, Jordan Simpson*, Igor Vouffo*, Omonike A Olaleye, Hamisu M Salihu	A Surveillance System for the Maternal and Child Health (MCH) Population During the COVID-19 Pandemic	Int J MCH AIDS DOI: 10.21106/ijma.411	2021	Published
Gabriella Tavera*, Deepa Dongarwar, Jason L Salemi, Oyinkansola Akindela*, Itohan Osazuwa*, Eyerusalem B Akpan*, Ugonna Okolie*, Marilynn Johnson*, Kiara K Spooner, Ubong I Akpan, Korede K Yusuf, Chidinma Chukwudum*, Hamisu M Salihu, Omonike A Olaleye	Diabetes in Pregnancy and Risk of Near-Miss, Maternal Mortality and Fetal Outcomes in the USA: a Retrospective Cross-Sectional Analysis	J Public Health (Oxf) DOI: 10.1093/pubmed/fdab117	2021	Published

Overall, 31 BCM–TSU students were either primary or co-authors in these publications. Over the same period, 20 BCM–TSU students received MCH internships in agencies/organizations; 22 students were offered admission into MCH-related graduate programs; and 80 students submitted abstracts and presented at local, regional or national conferences. Table 2 shows the list of publications from the BCM–TSU MCH LEAP program (updated to December, 2021).

## Discussion

Participants of the MCH LEAP who received research mentorship through the BCM–TSU MCHPTP reported improvements in theoretical knowledge and competency skills in the field of MCH research. Under-represented undergraduate students, who otherwise would not have had exposure to MCH training and experience in the research were able to achieve outstanding outcomes such as submitting and presenting abstracts and posters at national conferences as well as being able to draft, submit and publish in peer-reviewed journals. Through continuous mentorship from the faculty and staff and networking with like-minded MCH professionals in a variety of intellectually stimulating avenues, the majority of the students decided to advance their career in the same field, either by joining MCH-related or public health graduate programs or by obtaining internship and permanent job placements within the MCH workforce. Since 2006, the number of MCHPTPs has doubled, and this is a reflection of the needs resulting from workforce expansion over the decades.

In order to disseminate MCH research findings as well as conduct other health education activities collaboratively with community stakeholders, the students from the BCM–TSU program created an organization called Global Alliance for Maternal and Child Health (GLAM) in 2019, whose mission is to establish productive alliances and partnerships committed to the elimination of health disparities in MCH populations. This will be accomplished through community outreach and global engagement using evidence-based strategies (De Weger et al., 2018). GLAM’s objectives include—providing members the platform to develop MCH leadership and professional skills; offering collaborative opportunities to students and the community for MCH advancement and global engagement; educating communities about MCH-related prevention, treatment, and management services; promoting awareness, providing resources, and strengthening MCH research towards the elimination of health disparities, especially in underserved communities; and finally, increasing the proportion of underrepresented and underserved populations in MCH pipelines nationwide as well as the workforce. Therefore, the creation of GLAM is a testament to the degree to which BCM–TSU student engagement in MCH research activities coupled with acquisition of knowledge and skills have translated into action plans advocated by the students themselves, a welcome development that will contribute significantly to sustainability of current accomplishments. One limitation of this study is that we are unable to elaborate on the research outcomes from the other MCHPTP since each MCHPTP focused on a unique outcome and BCM–TSU was the only one with its focus on MCH research.

Over the years, the National Institute of General Medical Sciences (NIGMS) has strived to increase the number of underrepresented minorities participating in biomedical research by establishing the Division of Minority Opportunities in Research (National Research Council, 2003). Despite the initial target of eliminating longstanding disparities in the health status of racial and ethnic minority groups by 2010, there is still a lot of work to be done (Bozlak et al., 2022; Mishkin et al., 2021; National Research Council, 2003). Clinical research has shown that a patient’s race and ethnicity is highly correlated with their health outcomes. For example, it was found that up to 75% of Pacific Islanders are unable to convert the antiplatelet drug clopidogrel into its active form and are therefore, at higher risk for adverse outcomes following angioplasty; and without recruiting candidates from varied backgrounds, such nuances can be missed (Oh et al., 2015). When researchers and health care providers identify with and understand the population sub-group they are working with, the health outcomes for the target community tend to improve (Bouye et al., 2016; Phillips & Malone, 2014). When there are culturally representative researchers tending to the diverse MCH population, barriers such as fears of exploitation in medical research, financial constraints, competing demands of time, lack of access to information and comprehension about research, unique cultural and linguistic differences, fears of unintended outcomes, stigmatization, and health care discrimination that prevent minority population from participating in research could be overcome (Oh et al., 2015). Given the aforementioned advantages, an HRSA-funded MCH PTP represents a unique opportunity to strengthen the MCH workforce through early recruitment and training of underrepresented and underserved groups with a vision of attaining future goals that target elimination of health disparities in our society.

The research productivity of students in the six MCHPTPs is a predictor of the success of integrating MCH research experiences into MCH capacity building activities. MCH research helps to foster an environment where didactic and scholarly research is translated into real-world practice. The development of a diverse and highly skilled MCH pipeline promotes and strengthens research activities in areas of high priority such as addressing health disparities in MCH morbidity and mortality in the U.S. Expanding the pool of diverse MCH leaders and policymakers in the workforce may improve the ability to address current and emerging MCH-needs.

## Data Availability

Not applicable.
